# BioDecryptors, a Bioinformatics Escape Game for Undergraduate and Postgraduate Learners in Clinical Microbiology: Prospective Before-and-After Study

**DOI:** 10.2196/94592

**Published:** 2026-11-05

**Authors:** Sarah Dellière, Rémi Gschwind, Maud Salmona

**Affiliations:** 1 Université Paris Cité, Parasitology Mycology Department, Hôpital Bichat Claude Bernard, Assistance Publique – Hôpitaux de Paris (AP-HP), Paris, France; 2 Institut National de la Santé et de la Recherche Médicale (INSERM), Université Paris Cité, Paris, Île-de-France, France; 3 Université Paris Cité, Virology Department, Hôpital Saint-Louis, Assistance Publique – Hôpitaux de Paris (AP-HP), 1 av Claude Vellefaux, Paris, 75010, France, 33 142499480

**Keywords:** escape games, bioinformatics, clinical microbiology, medical education, gamification, microbial genomics

## Abstract

**Background:**

Bioinformatics is increasingly important in clinical microbiology with growing use of genomic sequencing, metagenomics, and phylogenetic analyses. However, introducing bioinformatics concepts and tools to medical learners can be challenging. Gamified educational approaches may allow introducing these concepts while supporting learner motivation.

**Objective:**

This study prospectively evaluated BioDecryptors, an educational escape game to introduce bioinformatics principles and tools in clinical microbiology, by assessing short-term changes in knowledge and self-reported motivation and evaluating participant satisfaction.

**Methods:**

We conducted a prospective before-and-after study between January and September 2025. Volunteers were recruited from undergraduate medical programs at Université Paris Cité and postgraduate programs or training activities for MDs and PharmDs at Université de Nantes, Université Paris-Saclay, and the French Society for Microbiology. Overall, 55 participants were included: 22 (40%) second-year medical students and 33 (60%) postgraduate MDs or PharmDs. Participants completed BioDecryptors in teams of 3 to 4 using versions adapted to their training level. Knowledge was assessed before and immediately after the activity using 2 distinct 10-item tests assessing the same predefined learning objectives. Motivation was assessed using a 12-item, 5-point Likert questionnaire covering 3 predefined domains, and satisfaction was assessed after the activity. Pre-post knowledge scores were compared using paired *t* tests or Wilcoxon signed-rank tests, as appropriate. Motivation items were analyzed using paired Wilcoxon signed-rank tests with Benjamini-Hochberg correction. Statistical significance was defined as a 2-sided *P*<.05.

**Results:**

Paired knowledge assessments were available for 50 participants. Mean knowledge scores increased from 7.0 (SD 2.0; 95% CI 6.4-7.5) before the activity to 7.9 (SD 2.0; 95% CI 7.4-8.5) afterward (*P*<.001). The mean change was 0.93 (SD 1.39; 95% CI 0.26-1.60) points among undergraduates and 0.83 (SD 1.21; 95% CI 0.38-1.27) points among postgraduates, with no statistically significant between-group difference (*P*=.79). Motivation increased for perceived usefulness (*P*=.04), willingness to learn (*P*=.01), understanding of bioinformatics in diseases (*P*=.002), and confidence in bioinformatics abilities (*P*=.002). Curiosity (*P*=.46) and interest in real-life applications (*P*=.33) did not significantly change. Among 50 satisfaction respondents, overall satisfaction was high (mean 4.7, SD 0.5; 95% CI 4.6‐4.9); 98% (48/49) of the respondents to the corresponding item indicated an interest in other escape game modules.

**Conclusions:**

Participation in BioDecryptors was associated with short-term increases in bioinformatics knowledge and self-reported motivation, together with high participant satisfaction. Unlike previously reported escape games focusing primarily on clinical or infection-control topics, BioDecryptors integrates hands-on bioinformatics analyses into a clinical microbiology scenario and offers versions adapted to undergraduate and postgraduate learners. Our findings provide preliminary evidence supporting the feasibility and acceptability of this approach as a complementary educational resource for introducing bioinformatics in health professions training. Controlled studies with longer-term assessments are needed to establish its comparative educational effectiveness and knowledge retention.

## Introduction

Medical educators are continuously seeking innovative methods to modernize teaching and enhance learner engagement, particularly since the COVID-19 pandemic, which accelerated the adoption of blended and virtual learning formats. Increasing evidence suggests that medical students prefer interactive, gamified approaches to learning over traditional didactic formats [1,2]. *Gamification*, the application of game mechanics to educational settings, has been shown to improve the achievement of learning objectives compared with conventional methods [3]. In this context, learners become active participants in their own education rather than passive recipients. One popular gamification strategy is the *escape room*, a team-based activity in which participants solve puzzles and riddles within a set time limit. Beyond fostering engagement and the acquisition of specific knowledge, escape games also promote teamwork and communication, both essential competencies in medical practice. Recent systematic reviews and meta-analyses have reported favorable effects of educational escape rooms on short-term knowledge acquisition, motivation, engagement, and satisfaction across health professions education [4,5]. However, their comparative benefit over conventional or other active learning approaches remains uncertain. The available literature is dominated by uncontrolled or quasi-experimental pre-post studies, with relatively few randomized or controlled comparisons, substantial heterogeneity in interventions and outcome measures, and limited assessment of long-term knowledge retention [5,6].

As medical knowledge rapidly evolves, curricula must adapt accordingly. The integration of bioinformatics into medical education has become increasingly necessary, reflecting the growing role of genomics, big data, and computational methods in clinical practice and research [7]. This is especially true in clinical microbiology, where modern diagnostics and outbreak investigations increasingly rely on molecular methods, including metagenomics and other data-driven techniques. For physicians in microbiology and infectious diseases, a foundational understanding of bioinformatics is critical, given its direct impact on diagnostic accuracy, laboratory efficiency, and public health surveillance [8]. Importantly, bioinformatics training does not always require advanced coding skills, as many user-friendly platforms, such as Galaxy (Galaxy Project) or web-based software tools [9,10], allow students to perform basic analyses. Moreover, the ability to independently explore and apply such tools is valuable, since formal training modules may be time-intensive and not always accessible. Nevertheless, bioinformatics combines biological, computational, and analytical concepts that may be challenging to introduce to learners with heterogeneous backgrounds and previous exposure to computational methods. This creates a need for educational approaches that contextualize bioinformatics within clinically relevant problems and provide opportunities to actively apply analytical concepts and tools [11,12].

Although introductory courses on analytical principles and bioinformatics tools have been incorporated into medical, pharmacy, and microbiology curricula, traditional approaches, including lectures and structured practicals, may not always sustain student engagement or promote long-term retention. Although escape games have increasingly been evaluated in health professions education, including for infectious diseases and infection prevention, their application to bioinformatics education in clinical microbiology remains largely unexplored. Recent escape games in infectious diseases have focused primarily on clinical knowledge and infection-control practices rather than bioinformatics analyses [13-15]. Conversely, gamified approaches to bioinformatics education have incorporated interactive digital tools, narrative e-learning, or game-based analogies but not the team-based escape game format [16-18]. To our knowledge, no previous study has evaluated a bioinformatics escape game in clinical microbiology across both undergraduate and postgraduate learners.

To address these challenges, we developed an educational *escape game* designed as an innovative, interactive, and immersive approach to introducing medical students, residents, and physicians at different training levels to the fundamentals of bioinformatics in clinical microbiology. The learning objectives of these sessions, which were delivered through autonomous play were to (1) provide students with an understanding of the principles underlying genomic, phylogenetic, and metagenomic analyses; (2) familiarize them with commonly used tools and data formats; and (3) offer hands-on experience with tasks such as chromatogram interpretation, database-based sequence comparison, sequence alignment, primer design, and phylogenetic reconstruction.

The primary objective of this study was to evaluate short-term changes in bioinformatics knowledge following participation in BioDecryptors. Secondary objectives were to assess changes in self-reported motivation toward bioinformatics and participant satisfaction. We also explored outcomes among undergraduate and postgraduate learners who completed versions of BioDecryptors adapted to their respective training levels.

## Methods

### Study Design and Setting

This prospective before-and-after educational study was conducted in France between January 2025 and September 2025 in undergraduate and postgraduate educational settings. Knowledge and motivation were assessed before and immediately after the educational activity, and satisfaction was assessed after the activity. The study did not include random allocation or a parallel control group.

### Escape Game Design

Students, working in teams of 3 to 4, were tasked with solving a viral pandemic scenario through several analytical steps: clinical and biological investigation, 16S ribosomal RNA (rRNA) and internal transcribed spacer (ITS) sequence analyses for bacteria and fungi, respectively, clinical metagenomics, polymerase chain reaction (PCR) primer design, and sequence reconstruction with phylogenetic analysis. All tools used were free and accessible online (Multimedia Appendix 1). Two versions of the escape game were developed according to the participants’ training levels. The undergraduate version, designed for medical students, included PCR primer design, whereas the postgraduate version, designed for MDs and PharmDs, included sequence reconstruction and phylogenetic analysis. Thus, undergraduate and postgraduate participants completed versions of BioDecryptors adapted to their respective levels of training, with differences in the complexity and content of the educational tasks. All images used in the activity were generated using AI (ChatGPT 3.0 image generator; OpenAI). The online tool Genially (Genially Web SL [19]) was used to provide interactive content.

### Participants and Recruitment

Participation was voluntary, and participants had the option to withdraw at any time. Participants were (1) second-year medical students at Université Paris Cité (Paris, France); (2) postgraduate MDs or PharmDs enrolled in a university diploma program in genomics applied to clinical microbiology at Université de Nantes (Nantes, France) or in next-generation sequencing technologies and bioinformatics at Université Paris -Saclay (Paris, France), and (3) postgraduate MDs or PharmDs aged <35 years who attended an introductory bioinformatics training day organized by the youth section of the French Society for Microbiology (Table S1 in Multimedia Appendix 1). Eligible participants were learners attending the educational sessions in which BioDecryptors was implemented, and no additional exclusion criteria were applied. All eligible learners attending these sessions were invited to participate. Convenience sampling was therefore used, and no random sampling was performed. All participants were recruited between January 2025 and September 2025.

### Ethical Considerations

This study was conducted as an evaluation of an educational intervention involving undergraduate and postgraduate learners from participating French universities and training programs. The activity formed part of the participating educational programs and did not involve medical interventions or the collection of personal health data. Under the applicable French regulatory framework, formal research ethics committee approval was not required [20,21].

All participants were informed about the study and provided informed consent before participation. Participation was voluntary, and participants could withdraw at any time.

Evaluation data were collected anonymously and used exclusively for educational research purposes. No directly identifying personal information was collected through the study questionnaires, and results are reported in aggregate form to protect participant privacy and confidentiality. Participants received no financial or other compensation for participating in the study. No images in the manuscript or supplementary materials contain identifiable participants; therefore, no image-specific consent was required.

### Evaluation Design

Participants completed a preactivity and postactivity motivation questionnaire (Multimedia Appendix 1) and a knowledge assessment. The 12-item motivation questionnaire was developed by the authors for this educational evaluation, drawing on the motivational principles of the Keller [22] attention, relevance, confidence, and satisfaction (ARCS) framework and adapting them to the context of bioinformatics education and the BioDecryptors activity. Items were rated on a 5-point Likert scale (1=“strongly disagree”; 5=“strongly agree”) and organized a priori into 3 domains: motivation and interest in bioinformatics, engagement and participation, and confidence in bioinformatics abilities. The questionnaire was not a direct implementation of a previously validated ARCS instrument and did not undergo formal factor or psychometric validation before the study. Knowledge acquisition was the primary outcome and was assessed using 2 distinct 10-item tests specifically developed by the authors for the BioDecryptors educational intervention (Multimedia Appendix 1), 1 administered before and the other immediately after the activity. Both assessments included multiple-choice, true or false, and single-answer items and were designed to assess the same predefined learning objectives related to sequencing analysis processes and bioinformatics tools covered during the activity. To assess the comparability of the 2 test versions, both assessments were previously administered sequentially to a separate sample of 11 clinical microbiology residents who did not participate in the escape game. Scores were 7.0 (95% CI 5.7-8.3) and 7.4 (95% CI 5.7-9.1), respectively (*P*=.26; Figure S1 in Multimedia Appendix 1). This preliminary assessment was intended to explore potential differences in test difficulty and did not constitute a parallel control group.

Secondary outcomes included attendance (the participation rate among second-year medical students relative to the overall course unit) and student satisfaction, measured with a Likert-scale questionnaire evaluating the playful aspect of the activity, the adequacy of its duration, perceived knowledge gain, level of autonomy, and quality of teamwork (Multimedia Appendix 1). Attendance data were obtained from attendance records for the undergraduate teaching sessions, whereas knowledge, motivation, and satisfaction data were obtained from the corresponding participant questionnaires and assessments. Training level was used for exploratory subgroup analyses. Because undergraduate and postgraduate participants differed in educational background and completed different versions of BioDecryptors, between-group comparisons were considered exploratory.

### Study Size

No a priori sample size or power calculation was performed. The study sample comprised eligible learners who agreed to participate in BioDecryptors during the predefined recruitment period. A total of 55 participants were included, comprising 22 undergraduate and 33 postgraduate participants.

### Statistical Analysis

Descriptive statistics were computed for all variables. Categorical variables were summarized as counts and percentages and continuous variables as means with 95% CIs or medians with IQRs, depending on the distribution of the data. Knowledge test scores were compared using paired *t* tests or Wilcoxon signed-rank tests, with between-group comparisons performed using Welch *t* test or Mann-Whitney *U* tests. Motivation scores were analyzed using paired Wilcoxon signed-rank tests (before vs after) and Mann-Whitney *U* tests (between groups). The internal consistency of each of the 3 predefined motivation domains was assessed using Cronbach α before and after the intervention. To account for multiple testing across the 12 pre-post motivation item comparisons, *P* values were adjusted using the Benjamini-Hochberg false discovery rate procedure. Correlations between baseline motivation and knowledge score improvement were assessed with Pearson or Spearman coefficients. Statistical significance was set at α=.05. Analyses were conducted in R (version 4.3.0; R Foundation for Statistical Computing).

Missing data were reported for each outcome, and paired analyses were restricted to participants with both preactivity and postactivity assessments. The missing completely at random (MCAR) assumption was assessed using the Little test. As a sensitivity analysis, missing knowledge and motivation data were imputed using multiple imputation by chained equations with predictive mean matching (50 imputed datasets), and pooled estimates were obtained using Rubin rules. Missing satisfaction data were not imputed because these data could not be linked to the other assessments. Effect sizes for pre-post changes were calculated using Cohen *d* for paired samples (*d*_z_) for knowledge scores and matched-pairs rank-biserial correlations for motivation items. Effect sizes are reported with 95% CIs.

## Results

A total of 55 students participated in the escape game: 22 (40%) undergraduate medical students and 33 (60%) postgraduate students, who played level 1 and level 2, respectively. Participant flow and the availability of data for each assessment are shown in Figure 1. The postactivity knowledge test was missing for 5 participants (9.1%), the postactivity motivation questionnaire for 7 (12.7%), and the satisfaction questionnaire for 5 (9.1%). The Little test did not reject the MCAR assumption (*χ*²_19_=21.4; *P*=.32). Multiple imputation yielded results consistent with the complete case analyses, with no change in the conclusions for knowledge or motivation outcomes. Complete paired knowledge assessments were available for 50 participants (31 postgraduate and 19 undergraduate). Overall, postactivity test scores increased significantly from 7.0 (SD 2.0; 95% CI 6.4‐7.5) to 7.9 (SD 2.0; 95% CI 7.4-8.5; *P*<.001; Figure 2A). When analyzed by subgroup, this improvement was significant for both undergraduate students (5.4, SD 1.4; 95% CI 4.7-6 vs 6.2, SD 1.5; 95% CI 5.5-6.9; *P*=.02) and for postgraduate students (8.1, SD 1.6 95% CI 7.5-8.6 vs 9.0, SD 1.4; 95% CI 8.5-9.5; *P*<.001; Figure 2B). The mean change was 0.93 (SD 1.39; 95% CI 0.26-1.60) points among undergraduates and 0.83 (SD 1.21; 95% CI 0.38-1.27) points among postgraduates. The mean difference in improvement between undergraduate and postgraduate participants was 0.11 points (95% CI 0.68-0.89; *P*=.79), providing no clear evidence of a between-group difference. Attendance was recorded for each teaching session (n=9) of the undergraduate medical student module. Attendance rates were significantly higher during the escape game sessions (n=2, 89.1%) than during the regular lecture sessions (n=7, 69.5%; *P*=.01).

**Figure 1.**
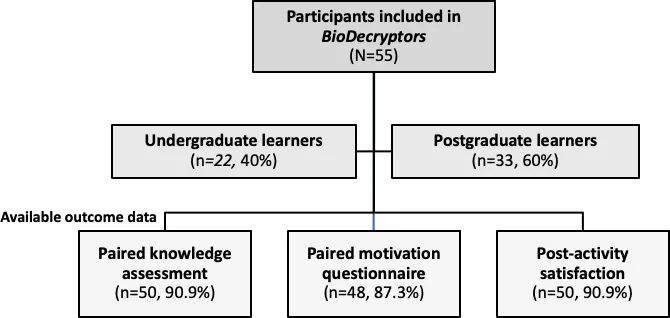
Participant flow throughout the study.

**Figure 2.**
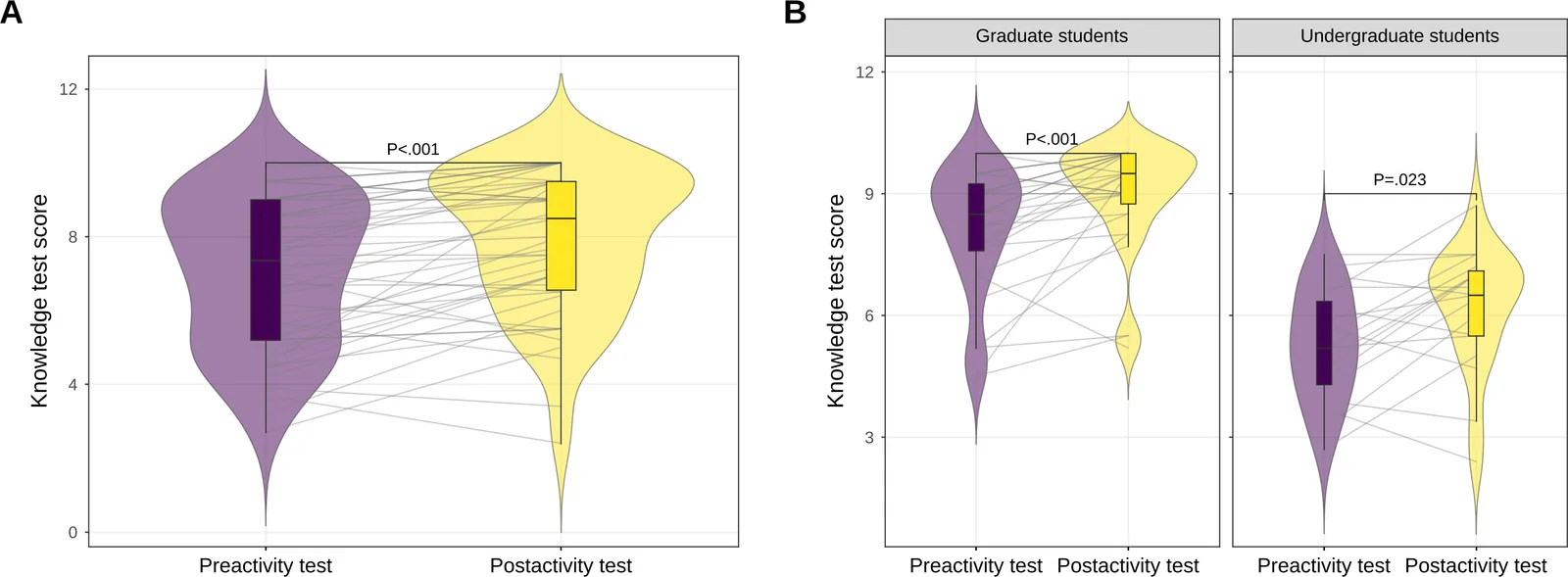
Preactivity and postactivity knowledge test scores (A) overall and (B) by training program following the BioDecryptors escape game.

Missing outcome data resulted from participants not completing the corresponding assessments or questionnaires rather than withdrawal from the BioDecryptors activity. Sample sizes therefore vary across outcomes.

Student perceptions and motivation toward bioinformatics were assessed across three domains: (1) motivation and interest in bioinformatics, (2) engagement and participation, and (3) confidence in bioinformatics abilities (Table 1). At baseline, postgraduate students reported significantly higher scores in the motivation and interest domain for *willingness to learn* (4.6, SD 0.9; 95% CI 4.2-4.9 vs 3.5, SD 1.0; 95% CI 3.1-3.9; *P*<.001) and *curiosity about applications* (4.7, SD 0.9; 95% CI 4.3-5.0 vs 4.1, SD 1.0; 95% CI 3.6-4.5; *P*=.02) than undergraduates. Perceived usefulness of *bioinformatics in medicine* did not differ significantly between groups (4.7, SD 0.9; 95% CI 4.4-5.0) vs 4.3, SD 1.0; 95% CI 3.8-4.7; *P*=.12). No significant differences were observed between groups in the engagement and participation domain, including *teamwork* (4.3, SD 1.0; 95% CI 3.9-4.6 vs 3.8, SD 1.0; 95% CI 3.4-4.3; *P*=.11), *interactive learning* (4.2, SD 1.1 95% CI 3.8-4.5 vs 4.5, SD 1.0; 95% CI 4.0-4.9; *P*=.30), *enjoyment of puzzles* (4.3, SD 0.9; 95% CI 4.0-4.6 vs 4.3, SD 1.1; 95% CI 3.8-4.8; *P*>.90), and *appreciation of real-life applications* (4.6, SD 0.9; 95% CI 4.3-4.9 vs 4.7, SD 0.9; 95% CI 4.3-5.1; *P*=.80). In contrast, postgraduate students reported consistently higher confidence in bioinformatics abilities, including *basic skills* (4.0, SD 1.0; 95% CI 3.7-4.3 vs 3.4, SD 0.8; 95% CI 3.0-3.7; *P*=.01), *understanding principles* (4.1, SD 0.9; 95% CI 3.8-4.4 vs 3.4, SD 0.8; 95% CI 3.1-3.8; *P*=.004), *confidence in their ability* (3.8, SD 1.0; 95% CI 3.4-4.1 vs 2.8, SD 1.1; 95% CI 2.3-3.3; *P*=.002), and *use of digital tools* (3.5, SD 1.7 95% CI 2.9-4.1 vs 1.6, SD 1.1 95% CI 1.1-2.1; *P*<.001).

Available data were as follows: preactivity scores and preactivity motivation (n=55; postgraduate student: n=33 and undergraduate: n=22), postactivity scores (n=50; postgraduate: n=31 and undergraduate: n=19), and postactivity motivation (n=48; postgraduate: n=29 and undergraduate: n=19).

**Table 1.** Comparison of preactivity and postactivity scores and motivation between graduate and undergraduate students.

Characteristics	Overall (N=55), mean (SD; 95% CI)	Graduate students (n=33), mean (SD; 95% CI)	Undergraduate students (n=22), mean (SD; 95% CI)	*P* value^a^
Test scores				
Preactivity score	7.0 (2.0; 6.4-7.5)	8.1 (1.6; 7.5-8.6)	5.4 (1.4; 4.7-6.0)	*<.001* ^b^
Postactivity score	7.9 (2.0; 7.4-8.5)	9.0 (1.4; 8.5-9.5)	6.2 (1.5; 5.5-6.9)	*<.001*
Motivation (preactivity score)^c^				
Motivation and interest in bioinformatics				
Perceived usefulness of bioinformatics in medicine	4.5 (1.0; 4.3-4.8)	4.7 (0.9; 4.4-5.0)	4.3 (1.0; 3.8-4.7)	.12
Willingness to learn more about bioinformatics	4.1 (1.1; 3.8-4.4)	4.6 (0.9; 4.2-4.9)	3.5 (1.0; 3.1-3.9)	*<.001*
Curiosity about applications of bioinformatics in medicine	4.4 (1.0; 4.1-4.7)	4.7 (0.9; 4.3-5.0)	4.1 (1.0; 3.6-4.5)	*.02*
Perceived contribution of bioinformatics to understanding diseases	4.0 (1.1; 3.7-4.3)	4.3 (1.1; 3.9-4.7)	3.6 (0.9; 3.2-4.0)	*.01*
Engagement and participation				
Enjoyment of teamwork	4.1 (1.0; 3.8-4.4)	4.3 (1.0; 3.9-4.6)	3.8 (1.0; 3.4-4.3)	.11
Preference for interactive learning	4.3 (1.0; 4.0-4.6)	4.2 (1.1; 3.8-4.5)	4.5 (1.0; 4.0-4.9)	.30
Enjoyment of puzzles and challenges	4.3 (1.0; 4.0-4.6)	4.3 (0.9; 4.0-4.6)	4.3 (1.1; 3.8-4.8)	>.90
Enjoyment of applying knowledge in real-life situations	4.6 (0.9; 4.4-4.9)	4.6 (0.9; 4.3-4.9)	4.7 (0.9; 4.3-5.1)	.80
Confidence in bioinformatics abilities				
Confidence in learning basic bioinformatics concepts	3.7 (1.0; 3.5-4.0)	4.0 (1.0; 3.7-4.3)	3.4 (0.8; 3.0-3.7)	*.01*
Ability to understand fundamental principles	3.8 (0.9; 3.6-4.1)	4.1 (0.9; 3.8-4.4)	3.4 (0.8; 3.1-3.8)	*.004*
Confidence in using biological data with digital tools	3.4 (1.1; 3.1-3.7)	3.8 (1.0; 3.4-4.1)	2.8 (1.1; 2.3-3.3)	*.002*
Prior experience with digital bioinformatics tools	2.7 (1.7; 2.3-3.2)	3.5 (1.7; 2.9-4.1)	1.6 (1.1; 1.1-2.1)	*<.001*
Motivation (postactivity score)^c^				
Motivation and interest in bioinformatics				
Perceived usefulness of bioinformatics in medicine	4.7 (0.7; 4.5-4.9)	4.8 (0.8; 4.5-5.1)	4.7 (0.6; 4.4-5.0)	.70
Willingness to learn more about bioinformatics	4.5 (0.8; 4.3-4.7)	4.7 (0.8; 4.4-5.0)	4.2 (0.7; 3.9-4.6)	*.04*
Curiosity about applications of bioinformatics in medicine	4.5 (0.9; 4.2-4.8)	4.7 (0.8; 4.4-5.0)	4.2 (1.0; 3.7-4.6)	*.048*
Perceived contribution of bioinformatics to understand diseases	4.4 (0.9; 4.2-4.7)	4.6 (0.9; 4.2-4.9)	4.3 (0.9; 3.8-4.7)	.30
Engagement and participation				
Enjoyment of teamwork	4.5 (0.7; 4.3-4.7)	4.5 (0.7; 4.2-4.7)	4.5 (0.6; 4.2-4.8)	.70
Preference for interactive learning	4.6 (0.6; 4.4-4.8)	4.5 (0.7; 4.3-4.8)	4.7 (0.6; 4.5-5.0)	.20
Enjoyment of puzzles and challenges	4.6 (0.7; 4.5-4.8)	4.6 (0.7; 4.3-4.9)	4.7 (0.6; 4.4-5.0)	.70
Enjoyment of applying knowledge in real-life situations	4.8 (0.6; 4.6-4.9)	4.7 (0.7; 4.5-5.0)	4.8 (0.4; 4.6-5.0)	.70
Confidence in bioinformatics abilities				
Confidence in learning basic bioinformatics concepts	4.1 (0.9; 3.9-4.4)	4.3 (0.8; 4.0-4.6)	3.9 (1.0; 3.4-4.4)	.20
Ability to understand fundamental principles	4.1 (0.8; 3.9-4.4)	4.2 (0.9; 3.9-4.6)	4.0 (0.8; 3.6-4.3)	.20
Confidence in using biological data with digital tools	4.1 (0.9; 3.9-4.4)	4.1 (0.9; 3.8-4.4)	4.2 (1.0; 3.7-4.7)	.70
Prior experience with digital bioinformatics tools	4.4 (1.1; 4.0-4.7)	4.3 (1.1; 3.8-4.7)	4.5 (1.1; 4.0-5.0)	.50

a *P* values are based on the Welch 2-sample *t* test.

b Statistically significant *P* values (*P*<.05) are shown in italics.

c Motivation items were rated on a 5-point Likert scale (1=“strongly disagree”; 5=“strongly agree”).

Following the activity, most of the baseline differences between undergraduate and postgraduate participants were no longer statistically significant. In the confidence domain, no statistically significant between-group differences were observed for *basic skills* (4.3, SD 0.8; 95% CI 4.0-4.6 vs 3.9, SD 1.1; 95% CI 3.4-4.4; *P*=.20), *understanding principles* (4.2, SD 0.9; 95% CI 3.9-4.6 vs 4.0, SD 0.8 95% CI 3.6-4.3; *P*=.20), *confidence in their ability* (4.1, SD 0.9 95% CI 3.8-4.4 vs 4.2, SD 1.0 95% CI 3.7-4.7; *P*=.70), and *use of digital tools* (4.3, SD 1.1 95% CI 3.8-4.7 vs 4.5, SD 1.1 95% CI 4.0-5.0; *P*=.50). However, 2 items in the motivation and interest domain remained significantly higher among postgraduate students: *willingness to learn* (4.7, SD 0.8 95% CI 4.4-5.0 vs 4.2, SD 0.7 95% CI 3.9-4.6; *P*=.04) and *curiosity about applications* (4.7, SD 0.8 95% CI 4.4-5.0 vs 4.2, SD 1.0; 95% CI 3.7-4.6; *P*=.048; Table 1).

Among all participants, several motivation scores increased significantly following the activity. In the *motivation and interest* category, perceived utility of bioinformatics in medicine (4.5, SD 1.0; 95% CI 4.3-4.8 vs 4.7, SD 0.7; 95% CI 4.5-4.9; adjusted *P*=.04), willingness to learn (4.1, SD 1.1; 95% CI 3.8-4.4 vs 4.5, SD 0.8; 95% CI 4.3-4.7; adjusted *P*=.01), and perceived contribution to understanding diseases (4.0, SD 1.1; 95% CI 3.7-4.3 vs 4.4, SD 0.9 95% CI 4.2-4.7; adjusted *P*=.002) all improved, while curiosity remained stable (4.4, SD 1.0; 95% CI 4.2-4.7 vs 4.5, SD 0.9; 95% CI 4.2‐4.8; adjusted *P*=0.46; Figure 3A). In the *engagement and participation* category, enjoyment of teamwork (4.1, SD 1.0; 95% CI 3.8-4.4 vs 4.5, SD 0.6; 95% CI 4.3-4.7; adjusted *P*=.02), interactive learning (4.3, SD 1.0; 95% CI 4.0-4.5 vs 4.6, SD 0.7; 95% CI 4.4-4.8; adjusted *P*=.04), and solving puzzles (4.3, SD 1.0; 95% CI 4.0-4.5 vs 4.6, SD 0.7; 95% CI 4.4-4.8; adjusted *P*=.02) increased, whereas appreciation of real-life applications did not change (4.6, SD 0.9 ; 95% CI 4.4-4.9 vs 4.8, SD 0.6; 95% CI 4.6-4.9; adjusted *P*=.33; Figure 3B). Finally, in the *confidence in bioinformatics abilities* category, students reported higher confidence in learning basic concepts (3.7, SD 1.0; 95% CI 3.5-4.0 vs 4.1, SD 0.9; 95% CI 3.9-4.4; adjusted *P*=.009), understanding fundamental principles (3.8, SD 0.9; 95% CI 3.6-4.1 vs 4.1, SD 0.8; 95% CI 3.9-4.4; adjusted *P*=.02), using digital tools for bioinformatics (2.7, SD 1.7; 95% CI 2.3-3.2 vs 4.4, SD 1.1; 95% CI 4.0-4.7; adjusted *P*<.001), and overall abilities (3.4, SD 1.1; 95% CI 3.1-3.7 vs 4.1, SD 0.9; 95% CI 3.9-4.4; adjusted *P*=.002; Figure 3C). Internal consistency was high for the motivation and interest in bioinformatics domain (preactivity: Cronbach α=0.91, 95% CI 0.86-0.94; postactivity: Cronbach α=0.92, 95% CI 0.87-0.95), good for engagement and participation (Cronbach α=0.81, 95% CI 0.72-0.88; Cronbach α=0.86, 95% CI 0.78-0.91), and acceptable to good for confidence in bioinformatics abilities (Cronbach α=0.74, 95% CI 0.61-0.84; Cronbach α=0.84, 95% CI 0.74-0.90). Effect sizes for pre-post changes in knowledge and motivation outcomes are reported in Table S2 in Multimedia Appendix 1.

**Figure 3.**
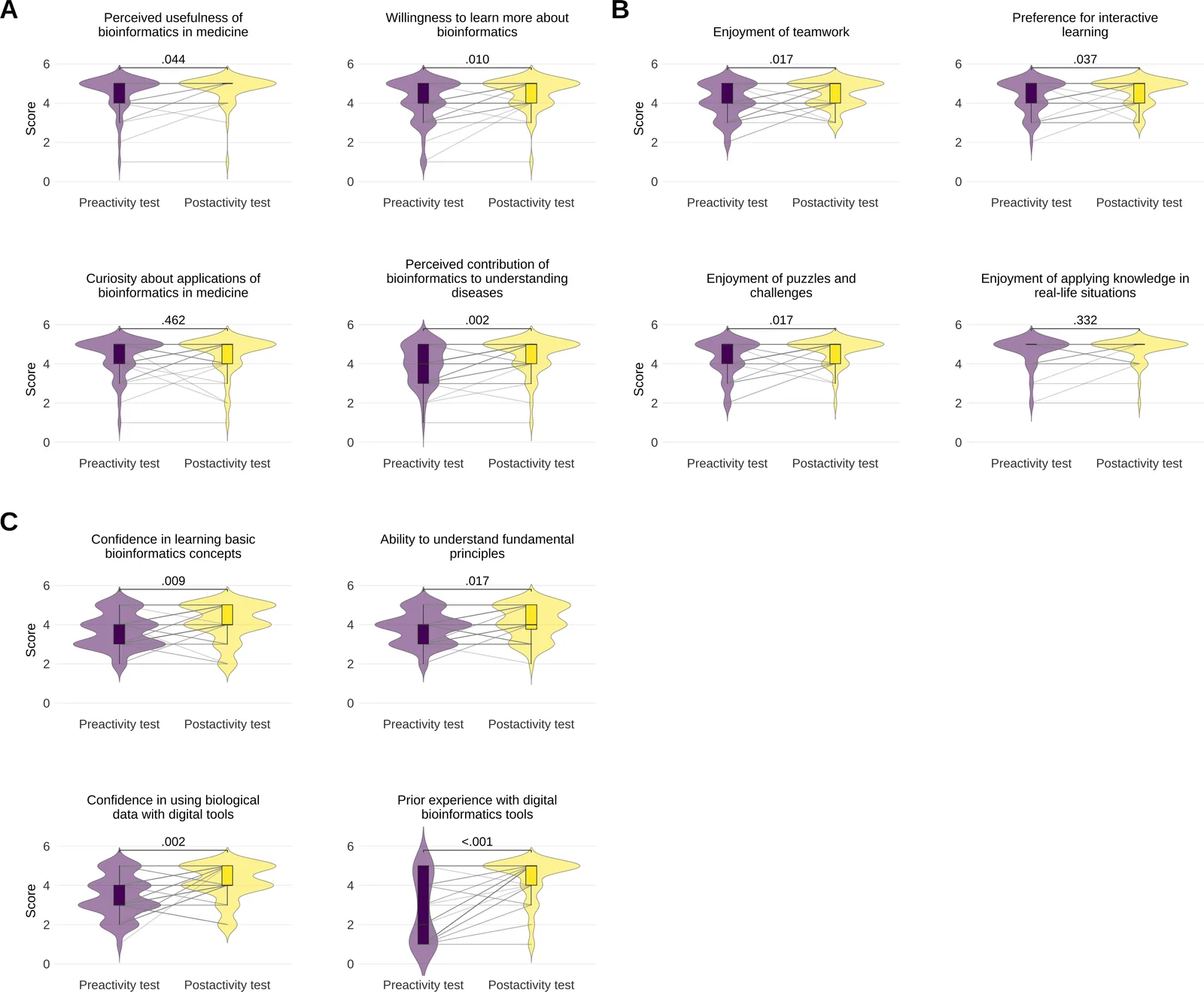
Student motivation for bioinformatics before and after the escape game. Violin plots show paired preactivity and postactivity responses to the 12-item questionnaire, rated on a 5-point Likert scale. Items are grouped into 3 predefined domains: (A) motivation and interest in bioinformatics, (B) engagement and participation, and (C) confidence in bioinformatics abilities. *P* values were calculated using paired Wilcoxon signed-rank tests and adjusted across the 12 item-level comparisons using the Benjamini-Hochberg procedure to control the false discovery rate. Lines connect paired observations.

We next examined whether baseline motivation was associated with learning gains. Correlation analyses between preactivity motivation scores and the difference in test scores (postactivity score–preactivity score) showed no significant associations for any item (all *P*>.10; Figure S2 in Multimedia Appendix 1).

Among the 50 respondents, overall satisfaction with the escape game was very high (4.7, SD 0.5; 95% CI 4.6-4.9), and the playful aspect obtained the highest mean score (4.9, SD 0.4; 95% CI 4.5-5.0). Perceived interest in the content was also highly rated (4.8, 95% CI 4.6-5.0), while clarity of instructions (4.6, SD 0.6; 95% CI 4.2-4.9) and time management (4.6, SD 0.6; 95% CI 4.2-4.8) were positively evaluated (Figure 4, Table 2). When comparing groups, postgraduate students provided slightly higher ratings than undergraduates. In particular, they rated the contribution of the activity to their learning significantly higher (4.8, SD 0.4; 95% CI 4.6-4.9 vs 4.4, SD 0.7; 95% CI 4.0-4.7; *P*=.03). Concerning overall satisfaction, the playful aspect, interest of the content, clarity of instructions, and time management, differences between postgraduate and undergraduate students were not statistically significant (Table 2). Finally, 98% (95% CI 88%-100%) of respondents expressed interest in participating in similar escape game activities in the future

**Figure 4.**
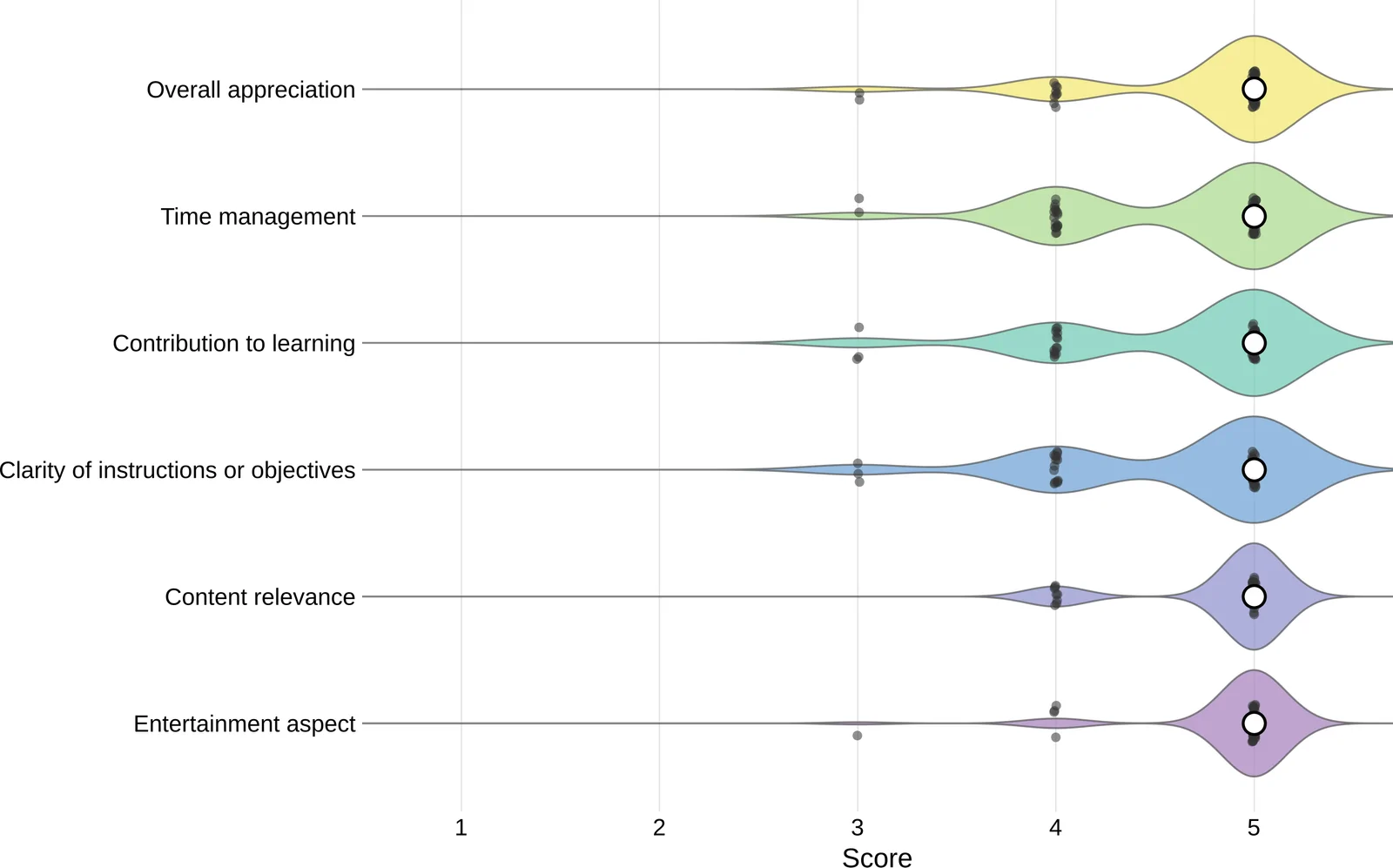
Student satisfaction with the BioDecryptors escape game. Distribution of responses on a 5-point Likert scale regarding overall appreciation, playful aspect, contribution to learning, content relevance, clarity of instructions or objectives, and adequacy of time management.

**Table 2.** Student satisfaction with the escape game activity^a^.

Characteristics	Overall (N=50)	Postgraduate students (n=291)	Undergraduate students (n=211)	*P* value^b^
Overall appreciation, mean (SD; 95% CI)	4.74 (0.53; 4.6‐4.9)	4.83 (0.38; 4.7‐5.0)	4.62 (0.67; 4.3‐4.9)	.30
Contribution to learning, mean (SD; 95% CI)	4.62 (0.60; 4.4‐4.8)	4.79 (0.41; 4.6‐4.9)	4.38 (0.74; 4.0‐4.7)	*.03*
Playful aspect, mean (SD; 95% CI)	4.88 (0.39; 4.8‐5.0)	4.97 (0.19; 4.9‐5.0)	4.75 (0.55; 4.5‐5.0)	.06
Content relevance, mean (SD; 95% CI)	4.84 (0.37; 4.7‐4.9)	4.90 (0.31; 4.8‐5.0)	4.76 (0.44; 4.6‐5.0)	.20
Clarity of instructions or objectives, mean (SD; 95% CI)	4.59 (0.61; 4.4‐4.8)	4.61 (0.50; 4.4‐4.8)	4.57 (0.75; 4.2‐4.9)	.70
Time management, mean (SD; 95% CI)	4.58 (0.57; 4.4‐4.7)	4.66 (0.48; 4.5‐4.8)	4.48 (0.68; 4.2‐4.8)	.40
Other escape game modules, n (%; 95% CI)				>.90
No	1 (2; 0.1%‐12%)	1 (3.6; 0.2%‐20%)	0 (0; 0.0%‐19%)	
Yes	48 (98; 88%‐100%)	27 (96; 80%‐100%)	21 (100; 81%‐100%)	

a Items were rated on a 5-point Likert scale (1=“strongly disagree”; 5=“strongly agree”).

b Wilcoxon rank sum test and Fisher exact test.

## Discussion

### Principal Findings

In this prospective before-and-after study, participation in the educational escape game BioDecryptors was associated with short-term increases in bioinformatics knowledge and self-reported motivation among undergraduate and postgraduate medical trainees. Beyond academic performance, participants reported a greater willingness to learn and an increased perception of the importance of bioinformatics in microbiology. Furthermore, among undergraduates, attendance was higher for escape game sessions than for regular lectures. Overall satisfaction was high, with most students highlighting the playful and engaging nature of the format, its contribution to learning, and the quality of teamwork it encouraged. Together, these findings suggest that BioDecryptors is a feasible and well-accepted format for introducing bioinformatics concepts and tools across different levels of training.

Escape games have been used in several medical fields such as radiology [23], surgery [24], internal medicine [25], and dermatology [26]. An escape room designed for emergency medicine residents at 2 different programs showed a significant increase in their preactivity vs postactivity quiz scores among participants in the escape room groups, whereas the lectures did not result in a statistically significant improvement [13]. Meta-analytic and systematic reviews confirm that escape rooms consistently increase student engagement, motivation, and teacher enthusiasm, with immediate postintervention gains in knowledge and skills [4,5]. However, most studies focus on short-term outcomes, and only a minority use longitudinal designs or randomized controlled trials to assess retention beyond the immediate postintervention period [4,5]. When longer-term follow-up is reported, the effect on declarative knowledge is generally modest, and may not exceed that of other active learning modalities such as simulation [27]. Yet, the experience may remain more enjoyable for both students and teachers.

For students, participation in the escape game was associated with increased knowledge scores, willingness to learn, and confidence in several bioinformatics abilities. The playful and interactive format provided opportunities to apply bioinformatics concepts to concrete tasks within a clinical scenario. Students’ feedback highlighted the originality, interactivity, and practical nature of the activity: “Very enjoyable and highly original!” and “It was very interesting to be confronted with a practical case. I understood many things! Thank you very much.” Another participant emphasized the clinical and applied dimension: “A highly interactive teaching approach that combines the application of clinical reasoning with learning new bioinformatics tools and understanding their relevance. Many thanks!” In line with these comments, both undergraduate and postgraduate students expressed high levels of satisfaction with the escape game. The activity was perceived as engaging, relevant, and applicable to their training, with postgraduates particularly emphasizing its contribution to their learning experience. These motivational findings may also be considered in light of self-determination theory. Ahmadi et al [28] identified teaching behaviors that may support students’ autonomy, competence, and relatedness, thereby fostering motivation and engagement. The interactive, collaborative, and challenge-based features of BioDecryptors may potentially engage some of these mechanisms by providing opportunities for active decision-making, progressive mastery of tasks, and peer interaction. However, these specific motivational mechanisms were not directly assessed in the present study and should therefore be considered potential explanations rather than demonstrated effects of the intervention.

From an instructor perspective, escape game–based learning may also offer potential pedagogical advantages. Previous studies have suggested that this format can provide opportunities to observe group dynamics and students’ reasoning processes and may facilitate interactions between instructors and learners [29]. In addition, once developed, an escape game scenario can potentially be reused and adapted to different educational contexts. However, instructor perceptions and teaching outcomes were not formally evaluated in the present study; therefore, these potential benefits cannot be inferred from our findings [29].

### Strengths and Limitations

This study has several strengths. It is, to our knowledge, the first to design and implement an educational escape game specifically aimed at introducing bioinformatics in medical training. It included students from different institutions and training programs, increasing the diversity of the study population. The evaluation combined multiple outcomes—knowledge acquisition, motivation, and satisfaction—providing a comprehensive assessment of short-term educational outcomes and learner experience.

Some limitations should be acknowledged. The main limitation of this study is its before-and-after design without a parallel control group, which limits causal inference. Although knowledge scores increased after participation, these changes cannot be attributed specifically to the escape game format and may also reflect exposure to the educational content or other contextual factors. The small sample of clinical microbiology residents who completed both knowledge assessments without participating in the escape game does not constitute a formal control group. Consequently, our findings should be interpreted as showing an association between participation in BioDecryptors and short-term changes in knowledge and self-reported motivation rather than demonstrating the educational superiority of the escape game format. In addition, the sample size was modest, and only immediate outcomes were assessed; long-term retention of bioinformatics knowledge and skills was not evaluated. Finally, satisfaction and motivation were assessed using self-reported questionnaires and may therefore be subject to social desirability or response bias.

Postactivity data were missing for up to 12.7% (7/55) of participants. Although the Little test did not reject the MCAR assumption and multiple imputation yielded consistent results, the small number of incomplete cases limits the assessment of the missing data mechanism, and nonresponse bias cannot be excluded.

The study population was also heterogeneous with respect to training level and prior exposure to bioinformatics. Undergraduate and postgraduate participants completed versions of BioDecryptors adapted to their respective levels, with differences in the complexity and content of the educational tasks. Consequently, between-group analyses were exploratory and cannot establish whether the intervention had comparable effects across training levels. Rather, the findings suggest that the BioDecryptors format can be adapted for use with learners at different stages of training, although its effectiveness within each educational level should be evaluated independently in adequately powered studies.

A further limitation concerns the assessment instruments. Although the 2 10-item knowledge tests were designed to assess the same learning objectives and their comparability was explored in 11 clinical microbiology residents, they did not undergo formal psychometric validation, and differences in difficulty between the 2 versions cannot be excluded. Similarly, the motivation questionnaire was developed for this educational evaluation and, although internal consistency was acceptable to high across the predefined domains, it did not undergo formal factor analysis or psychometric validation. These limitations, together with the modest absolute improvement in knowledge scores, should be considered when interpreting the findings. Future studies should use validated assessment instruments and include delayed assessments to evaluate knowledge retention.

Future work should expand to larger cohorts across additional institutions and explore longer-term outcomes, including knowledge retention and the transferability of skills to clinical or research contexts. Adaptation of this gamified approach to other areas of microbiology and biomedical sciences could further extend its educational impact and confirm its value as a versatile and engaging teaching tool.

### Conclusions

In this prospective before-and-after study, participation in BioDecryptors was associated with short-term increases in bioinformatics knowledge and self-reported motivation, together with high participant satisfaction. The principal innovation of BioDecryptors lies in combining a collaborative escape game with hands-on bioinformatics analyses embedded in a clinically relevant microbiology scenario. Unlike previously reported escape games in infectious diseases that have primarily focused on clinical knowledge or infection prevention, BioDecryptors introduces learners to bioinformatics concepts and tools through versions adapted to undergraduate and postgraduate training levels. These findings support the feasibility and acceptability of this approach as a complementary educational resource for introducing bioinformatics in clinical microbiology. However, the absence of a parallel control group prevents attribution of the observed changes specifically to the escape game format, and the different versions completed by undergraduate and postgraduate learners preclude conclusions about comparable effectiveness across training levels. Future controlled studies with validated assessments and longer-term follow-up are needed to evaluate educational effectiveness, knowledge retention, and the transfer of skills to clinical or research settings.

## Supplementary material

10.2196/94592 Multimedia Appendix 1 Educational program descriptions, BioDecryptors game design, motivation and engagement questionnaire, knowledge tests, game evaluation questionnaire, effect sizes, and additional analyses of knowledge and motivation outcomes.

## Abbreviations

ARCS: attention, relevance, confidence, and satisfaction
MCAR: missing completely at random
PCR: polymerase chain reaction
rRNA: ribosomal RNA
