# Supplementary material for: BioDecryptors, a Bioinformatics Escape Game for Undergraduate and Postgraduate Learners in Clinical Microbiology: Prospective Before-and-After Study
Source: JMIR Serious Games. 2026 Nov 5;14:e94592. doi: 10.2196/94592 (PMC13739269; doi:10.2196/94592)
Supplement: Supplementary file 1 [file games-v14-e94592-s001.docx]

**Supplementary Materials**

**Table S1. Description of educational programs integrating the escape game activity**

| Faculty  Organisation | Level | Course | Course time | Target public | Number of participants |
| --- | --- | --- | --- | --- | --- |
| Université de Paris | Undergraduate | Research initiation program: new methods in microbiology | 1 semester | 2nd year medical students | 22 |
| Université de Nantes | Postgraduate | University diploma in genomics applied to clinical microbiology | 1 year | MD, PharmD | 11 |
| Université Paris-Saclay | Postgraduate | University diploma in next-generation sequencing (NGS) and bioinformatics | 1 year | MD, PharmD, biologists or engineers | 11 |
| Société Française de Microbiologie | Postgraduate | Introductory day in bioinformatics applied to microbiology | 1 day | Young (<35 years old) clinical microbiologists | 11 |

**Table S2. Effect sizes for pre-post changes in knowledge and motivation outcomes.**

| **Outcome** | **n** | **Effect size (95% CI)** |
| --- | --- | --- |
| **Knowledge score** |  |  |
| *Overall* | 50 | Cohen’s *d*z = 0.68 (0.37 to 0.99) |
| *Undergraduate* | 19 | Cohen’s *d*z = 0.67 (0.16 to 1.16) |
| *Postgraduate* | 31 | Cohen’s *d*z = 0.68 (0.29 to 1.07) |
| **Motivation and interest in bioinformatics** |  |  |
| *Bioinformatics is useful in medicine* | 48 | *r*rb = 0.83 (0.33 to 1.00) |
| *Willingness to learn more about bioinformatics* | 48 | *r*rb = 0.82 (0.47 to 1.00) |
| *Curiosity about applications of bioinformatics in medicine* | 48 | *r*rb = 0.20 (−0.35 to 0.71) |
| *Bioinformatics helps understand diseases* | 48 | *r*rb = 0.79 (0.51 to 1.00) |
| **Engagement and participation** |  |  |
| *Enjoys teamwork learning* | 48 | *r*rb = 0.64 (0.23 to 0.94) |
| *Preference for interactive learning* | 48 | *r*rb = 0.64 (0.12 to 1.00) |
| *Enjoys puzzles and challenges* | 48 | *r*rb = 0.61 (0.21 to 0.93) |
| *Enjoys applying knowledge in real-life situations* | 48 | *r*rb = 0.42 (−0.43 to 1.00) |
| **Confidence in bioinformatics abilities** |  |  |
| *Feels capable of learning basic bioinformatics* | 48 | *r*rb = 0.57 (0.24 to 0.84) |
| *Ability to understand fundamental principles* | 48 | *r*rb = 0.57 (0.19 to 0.86) |
| *Confidence in using biological data with digital tools* | 48 | *r*rb = 0.67 (0.38 to 0.87) |
| *Prior experience with digital bioinformatics tools* | 47 | *r*rb = 0.94 (0.82 to 1.00) |

**Effect sizes for knowledge scores are reported as Cohen’s d for paired samples (dz). Motivation effect sizes are reported as matched-pairs rank-biserial correlations (rrb), with positive values indicating higher post-activity ratings. Rank-biserial 95% CIs were obtained using percentile bootstrap with 5000 resamples.**

**Figure S1. Pre- and post-activity knowledge test scores of clinical microbiology residents (n=11) who did not participate in the Escape Game**

**Figure S2. Correlation between baseline motivation items and improvement in test scores (post–pre).**
Scatterplots display individual pre-activity motivation scores (x-axis) and the difference in test performance between post- and pre-activity assessments (y-axis). Each panel represents one motivation item. Blue lines indicate linear regression fits with 95% confidence intervals. No significant correlations were observed for any item (all p > 0.1)

**Appendix I. BioDecryptor game design**

**Part I**

A. **Situation :**

Students are given the following clinical case.

**Mr. Z, a 45-year-old man, is hospitalized for fever and confusion.**

**Past medical history:** appendectomy and hypertension.

**Lifestyle:** works in the tourism industry.

His first consultation dates back to February, when he presented to the emergency department with unexplained fever. Antibiotic therapy had no effect. He now returns on March 6, in a state of marked confusion: his speech is incoherent, and he is particularly aggressive. He is barely dressed, wearing only a torn T-shirt. His wife appears frightened and keeps her distance, but has nevertheless accompanied him and remains at his bedside when your team enters the room.

B. **Task**

Communication with the patient, the family, and the hospital staff to request additional tests is carried out via WhatsApp

Students are expected to :

- Ask details about patients history
- Ask for tests and radiology exam
- Ask to perform a clinical exam (interactive online exam)

C. **Mandatory findings to unlock next step**

Student asked :

- About travel history and found out he travel to Kirghizistan where he visited caves
- Ordered a cerebral MRI
- Ordered a CSF exam
- Ordered multiplex PCR on CSF for usual micro-organisms

**Part II**

A. **Situation**

After one week of care, your patient shows no improvement. Between a seizure and a delirium episode, he bites the nurse who was attempting to sedate him.

The patient dies before you can establish the diagnosis.

What would you generally propose to do, and how would you proceed with the remaining samples at your disposal?

B. **Mandatory answer to unlock next step**

16S RNA (and ITS) sequencing on CSF leftover or cerebral biopsy performed at autopsy

C. **Task**

Analyze chromatogram .ab1 file and perform a BLAST alignment

 Bioinformatic tool :

- Teal: <https://www.gear-genomics.com/teal/>
- Blast: <https://blast.ncbi.nlm.nih.gov/Blast.cgi>

D. **Mandatory finding to unlock next step**

RNA 16S: *Staphylococcus epidermidis*

ITS: *Malassezia* sp.

Conclusion: most likely a contamination from skin microbiota and asks to perform clinical untargeted metagenomic

**Part III**

A. **Situation**

The nurse who was bitten has not returned to work for a week and has given no news.
Doctors from several countries are reporting similar cases. The emergency departments are filling up and the population is panicking. The ECDC has issued the highest alert in order to identify the pathogen responsible for this pandemic. You are one of the few centers with a metagenomics platform. All eyes are on you.

Are you truly the team you claim to be?

B. **Task to unlock next step**

Reorganise clinical metagenomic steps on an interactive platform

<https://view.genially.com/65cdda1c2f05ac0014a8c9aa/interactive-content-etapes-sequencage>

C.**Situation**

This is your last chance to determine what the sample contains and to understand whether one of the detected microorganisms could be responsible for the outbreak. How could this patient have been infected in the first place...?

The sample has been processed, but the bioinformatician has disappeared. His computer is still connected to Galaxy, and you find some scribbled notes left on his desk.

Students receive .fastq file

Bioinformatic tool :

- <https://galaxy.pasteur.fr/>

D. **Mandatory finding to unlock next step**

Aravan Lyssavirus identified in the sample data set.

**E. Extra fun task**

“You have discovered the new pathogen responsible for the pandemic. Take a few minutes to savor this victory—and give it a name!

Quickly inform the ECDC by email of your choice, indicating the names of the researchers who contributed to this discovery.

Thanks to this breakthrough, the world’s leading immunology and pharmaceutical teams will now be able to work on developing a vaccine”

**Part IV – Level 1**

A. **Situation**

Outside, chaos reigns. The number of infected people is increasing day by day. Some healthcare staff are deserting, but your team remains fully committed.

Thanks to your discovery, the immunologists have managed to produce a vaccine! It can now be deployed to help stop the pandemic.

There is one problem: if the vaccine is administered to someone already infected, it worsens the disease and makes the patient even more aggressive. It is therefore crucial to ensure that patients are not infected before giving them the vaccine.

The ECDC is asking you to **develop a rapid test, a PCR, to quickly identify the pathogen in patients**. They are also sending you 5 patient samples to test urgently. Please return the results to the ECDC.

B. **Mandatory finding to unlock next step**

Students receive :

- A protocol for PCR design with hints
- The sequence on which to design primers
- .txt files with sequences (positive and negative) samples to test the primers

Bioinformatic tools :

- Primer Blast : <https://www.ncbi.nlm.nih.gov/tools/primer-blast/>
- In silico PCR software: <http://insilico.ehu.es/user_seqs/>

Expected results : they identify correctly positive and negative sample

C. **The end**

Your PCR works. The vaccine has been deployed to the entire non-infected population, who are now protected against the virus.
The surviving infected individuals have been placed in quarantine while awaiting the development of a curative treatment.

**Part IV – Level 2**

A. **Situation**

Outside, chaos reigns. The number of infected people continues to rise every day. While some medical staff choose to abandon their posts, your team remains united and ready to face the challenge. Your new mission is to **fully sequence the virus and analyze its phylogenetic relationships to determine its origin.** This crucial step will also enable other teams to develop effective treatments and vaccines. The ECDC urgently requests that you provide the complete sequence of the virus and its phylogenetic analysis. You know it belongs to the Lyssavirus family—but what is the specific strain? It is time to get to work.

B. **Mandatory finding to unlock next step**

Students receive :

- A phylogenetic analysis protocol to identify the new virus with hints
- Virus reads in .fastq
- GenBank reference sequence list
- A publication on Phylogeography of Lyssaviruses

Bioinformatic tools

- Galaxy : <https://usegalaxy.eu/>
- Itol : <https://itol.embl.de/>

C. **The end**

Congratulations, you have characterized the strain! Thanks to your efforts, a vaccine has been developed and rapidly deployed among the non-infected population, who are now protected against the virus. The infected survivors have been placed in quarantine while a curative treatment is being developed

# **Appendix II. Motivation and Engagement Questionnaire**

Instructions: Please indicate your level of agreement with the following statements by selecting a response between 1 (strongly disagree) and 5 (strongly agree).

## **Motivation and Interest in Bioinformatics**

| No. | Item | Agreement Level (1–5) |
| --- | --- | --- |
| 1 | I believe bioinformatics is a useful discipline in medicine. | 1 2 3 4 5 |
| 2 | I would like to learn more about bioinformatics. | 1 2 3 4 5 |
| 3 | I am curious to understand how bioinformatics tools can be applied in medicine. | 1 2 3 4 5 |
| 4 | I think bioinformatics can help me better understand certain diseases. | 1 2 3 4 5 |

## **Engagement and Participation**

| No. | Item | Agreement Level (1–5) |
| --- | --- | --- |
| 5 | I enjoy learning by working in a team. | 1 2 3 4 5 |
| 6 | I prefer interactive learning methods rather than traditional lectures. | 1 2 3 4 5 |
| 7 | I enjoy solving puzzles and challenges as a way to learn. | 1 2 3 4 5 |
| 8 | I like applying my knowledge in practical situations. | 1 2 3 4 5 |

## **Confidence in Bioinformatics Abilities**

| No. | Item | Agreement Level (1–5) |
| --- | --- | --- |
| 9 | I feel capable of learning the basics of bioinformatics. | 1 2 3 4 5 |
| 10 | I believe I can understand the fundamental principles of bioinformatics tools. | 1 2 3 4 5 |
| 11 | I am confident in my ability to use biological data with digital tools. | 1 2 3 4 5 |
| 12 | I have already used a digital tool to analyze biological data (e.g., BLAST, medical databases). | 1 2 3 4 5 |

# **Appendix III. Knowledge tests**

# A. **Pre-Test Questionnaire**

Instructions: Please answer the following questions. Multiple-choice questions may have one or more correct answers, and short-answer questions should be answered in your own words.

1. Q1 [MCQ] Clinical metagenomics using next-generation sequencing (mNGS):

- Identifies microorganisms in a given sample
- Targets only eukaryotic microorganisms
- Requires clinical and biological interpretation to provide a result
- Contamination is a major issue

2. Q2 [MCQ] Sanger sequencing of a taxonomic marker in a sample:

- May be useful in samples containing a microbial flora
- Is analyzed through chromatogram reading
- Can identify a microorganism that would not grow in culture
- Can simultaneously identify multiple microorganisms

3. Q3 [MCQ] Regarding the chromatogram below:

- The sequence can be aligned with databases to identify the closest match
- It is a sequence obtained from Sanger sequencing
- The sequence contains double peaks
- It may come from a mixture of two microorganisms

4. Q4 [MCQ] The 'bootstrap' method used in building a phylogenetic tree:

- Is a statistical method measuring branch robustness
- Artificially increases the number of sequences
- Applies only to protein sequences
- Is unnecessary if the sequences are perfectly aligned

5. Q5 [SCQ] Among these tools, which can be used to compare NGS sequences to a taxonomic database?

- Kraken2
- FastQC
- BWA
- Trimmomatic

6. Q6 [SCQ] After receiving your NGS sequencing data, what is your first step?

- Convert the file format
- Align sequences with a taxonomic database
- Check sequence quality and filter out low-quality reads
- Deplete human genome sequences

7. Q7 [SCQ] What type of file is obtained after NGS sequencing?

- FASTQ file
- FASTA file
- BAM file
- Chromatogram

8. Q8 [Short Answer] Cite a bacterial or fungal taxonomic marker.
9. Q9 [Short Answer] Cite a software that allows NGS sequence analysis with a graphical interface.
10. Q10 [SCQ] The following image is a final output file of:

- NGS metagenomics analysis
- Sanger classification analysis
- Proteomics analysis by mass spectrometry
- NGS quality control analysis

# B. **Post-Test Questionnaire**

Instructions: Please answer the following questions. Multiple-choice questions may have one or more correct answers, and short-answer questions should be answered in your own words.

11. Q1 [MCQ] Clinical metagenomics using next-generation sequencing (mNGS):

- A. Identifies microorganisms in a clinical sample without culture
- B. Enables detection of new viruses
- C. Requires bioinformatics analysis
- D. Requires primer design beforehand

12. Q2 [MCQ] When using Sanger sequencing to identify a microorganism in a clinical sample, it is necessary to:

- A. Target a conserved gene
- B. Clean FASTQ files before analysis
- C. Visualize a chromatogram
- D. Have a sample containing at least three microbial species

13. Q3 [SCQ] The image below is a final output file of:

- Bioinformatics analysis of Sanger sequencing
- Bioinformatics analysis of NGS sequencing
- Multiplex PCR
- Simplex PCR

14. Q4 [SCQ] What does a 'clade' represent in a phylogenetic tree?

- A. A group of organisms or sequences sharing a common ancestor
- B. A systematic error in alignment
- C. A set of genes encoding the same protein
- D. A non-coding region of the genome

15. Q5 [Ordering] Put the steps of clinical metagenomics bioinformatics analysis in the correct order (number 1–4):

- Data visualization
- Human genome depletion
- Alignment of sequences with a taxonomic database
- Quality control and filtering of sequences

16. Q6 [MRQ] The following graph shows that:

- These NGS sequences are of good quality
- These Sanger sequences are unreadable
- These NGS sequences are contaminated
- These Sanger sequences show contamination of the sample

17. Q7 [SCQ] Kraken2 is a tool that allows you to:

- Filter NGS sequences based on quality
- Visualize a chromatogram
- Align sequences to a taxonomic database
- Deplete sequences belonging to the human genome

18. Q8 [SCQ] What types of files are generated after Sanger sequencing?

- A. FASTQ
- B. BAM
- C. Chromatogram
- D. FASTA

19. Q9 [Short Answer] Which genetic marker can be used to identify bacteria or fungi?
20. Q10 [SCQ] A student wants to analyze metagenomic data using a bioinformatics pipeline with a graphical interface. Which tool is the most suitable?

- Galaxy
- R Studio
- PowerPoint
- Primer 3

**Appendix IV. Escape Game Evaluation Questionnaire**

**Instructions:** For each aspect of the escape game, please indicate your appreciation by ticking the appropriate box.

| **Aspect** | **Very Dissatisfied** | **Dissatisfied** | **Neutral** | **Satisfied** | **Very Satisfied** |
| --- | --- | --- | --- | --- | --- |
| Your overall appreciation of the escape game | ☐ | ☐ | ☐ | ☐ | ☐ |
| The contribution of the escape game to your learning | ☐ | ☐ | ☐ | ☐ | ☐ |
| The entertaining aspect of the escape game | ☐ | ☐ | ☐ | ☐ | ☐ |
| The relevance of the escape game content | ☐ | ☐ | ☐ | ☐ | ☐ |
| The clarity of the instructions and objectives of the escape game | ☐ | ☐ | ☐ | ☐ | ☐ |
| The time management of the escape game | ☐ | ☐ | ☐ | ☐ | ☐ |

**Additional Questions**

1. Would you like to see more lab sessions (practical work) in the form of escape games in other modules?
   - ☐ Yes
   - ☐ No
2. Open comments regarding the escape game:
   .......................................................................................
   .......................................................................................
